# DNA methylation gene-based models indicating independent poor outcome in prostate cancer

**DOI:** 10.1186/1471-2407-14-655

**Published:** 2014-09-06

**Authors:** Nataša Vasiljević, Amar S Ahmad, Mangesh A Thorat, Gabrielle Fisher, Daniel M Berney, Henrik Møller, Christopher S Foster, Jack Cuzick, Attila T Lorincz

**Affiliations:** Centre for Cancer Prevention, Wolfson Institute of Preventive Medicine, Barts and The London School of Medicine, Queen Mary University of London, London, EC1M 6BQ UK; Molecular Oncology Centre, Barts Cancer Institute, Queen Mary University of London, London, EC1M 6BQ UK; King’s College London, Cancer Epidemiology and Population Health, London, SE1 9RT UK; HCA International Pathology Laboratories, 2-22 Capper Street, London, WC1E 6JA UK

**Keywords:** DNA methylation, Prostate cancer, Progression biomarkers, Watchful waiting, Pyrosequencing

## Abstract

**Background:**

Prostate cancer has a variable clinical behaviour with frequently unpredictable outcome. DNA methylation plays an important role in determining the biology of cancer but prognostic information is scanty. We assessed the potential of gene-specific DNA methylation changes to predict death from prostate cancer in a cohort of untreated men in the UK.

**Methods:**

This was a population-based study in which cases were identified from six cancer registries in Great Britain. DNA was extracted from formalin-fixed paraffin wax-embedded transurethral prostate resection tissues collected during 1990-96 from men with clinically-localised cancer who chose not to be treated for at least 6 months following diagnosis. The primary end point was death from prostate cancer. Outcomes were determined through medical records and cancer registry records. Pyrosequencing was used to quantify methylation in 13 candidate genes with established or suggested roles in cancer. Univariate and multivariate Cox models were used to identify possible predictors for prostate cancer-related death.

**Results:**

Of 367 men, 99 died from prostate cancer during a median of 9.5 years follow-up (max = 20). Univariately, 12 genes were significantly associated with prostate cancer mortality, hazard ratios ranged between 1.09 and 1.28 per decile increase in methylation. Stepwise Cox regression modelling suggested that the methylation of genes *HSPB1*, *CCND2* and *DPYS* contributed objective prognostic information to Gleason score and PSA with respect to cancer-related death during follow-up (p = 0.006).

**Conclusion:**

Methylation of 13 genes was analysed in 367 men with localised prostate cancer who were conservatively treated and stratified with respect to death from prostate cancer and those who survived or died of other causes. Of the 13 genes analysed, differential methylation of *HSPB1*, *CCND2* and *DPYS* provided independent prognostic information. Assessment of gene-methylation may provide independent objective information that can be used to segregate prostate cancers at diagnosis into predicted behavioural groups.

**Electronic supplementary material:**

The online version of this article (doi:10.1186/1471-2407-14-655) contains supplementary material, which is available to authorized users.

## Background

Prostate cancer is the most common malignancy in men but a significant proportion of the cases are essentially harmless and will not result in morbidity or death if left untreated. Currently the best-available prognostic tool for routine management is Gleason score [1]. Nevertheless histopathology has some limitations such as intra- and inter-observer variability in grading [2] and for needle biopsies there is additional variability due to difficulty in targeting cores precisely to the cancerous areas. These sources of variability lead to quite large differences in the accuracy of diagnosis and prognosis. Testing serum for prostate specific antigen (PSA) has improved early detection and is an increasingly used screening tool, however, its poor specificity in combination with absence of a highly accurate prognostic tool may lead to increased numbers of invasive examinations and biopsies resulting in unnecessary treatment with risk of morbidity [3–5]. Therefore there is an urgent need for standardised quantifiable molecular biomarker assays to improve disease stratification and subsequent management [6].

DNA methylation (DNAme) is important for normal development in higher organisms. In the human genome, the majority of CpG dyads have similar patterns of methylation in normal and cancerous tissues. However, CG rich regions (so-called CpG islands) covering the promoters and first exons of over half of human genes often show highly variable methylation, which is considered of regulatory importance [7–9]. Abnormal DNAme contributes to the occurrence and progression of prostate cancer [10, 11]. Development of methylation assays to diagnose and/or predict disease outcomes in cancer patients undergoing active follow-up with minimal intervention is topical [12, 13]. In prostate cancer, numerous hypermethylated genes have been found, with *GSTP1*, *APC1* and *RARB* amongst the most frequently reported [14], and hitherto mainly assessed for diagnostic purposes. The few studies focusing on the prognostic value of methylation generally use time to biochemical recurrence after surgical treatment as the primary endpoint, which does not accurately estimate the potential of the cancer in terms of risk of death if left untreated [15–17]. Therefore, the primary purpose of this study was to explore the hypothesis that methylation testing of specific genes in men with untreated clinically-localised prostate cancer contributes objective information with respect to prostate cancers that will lead to death during follow-up. The principal objective was to assess the univariate prognostic biomarker potential of DNA methylation of 13 individual genes and multivariate combinations of genes, by analysing the association between methylation and death from prostate cancer as the primary endpoint. The secondary objective was to determine whether methylation-status improves prognostic value of current clinical reference variables (Gleason score and PSA) and finally to investigate mortality predictions of models fitted with variables that can be measured in serum (i.e. methylation and PSA). Candidate genes *GSTP1, APC, RARB, CCND2, SLIT2, SFN, SERPINB5, MAL, DPYS, TIG1, HIN1, PDLIM4* and *HSPB1* were investigated because they were earlier reported to be associated with the diagnosis or prognosis of prostate cancer in addition to a variety of other cancers [18–23].

Univariate analysis showed that genes assessed individually were only modest predictors of death from prostate cancer. However, multivariate analysis revealed that methylation of *DPYS*, *CCND2* and *HSPB1* together added a substantial amount of prognostic information not captured by any other measure and therefore may be useful for improvement of prostate cancer management.

## Methods

### Study population

**Figure 1:**
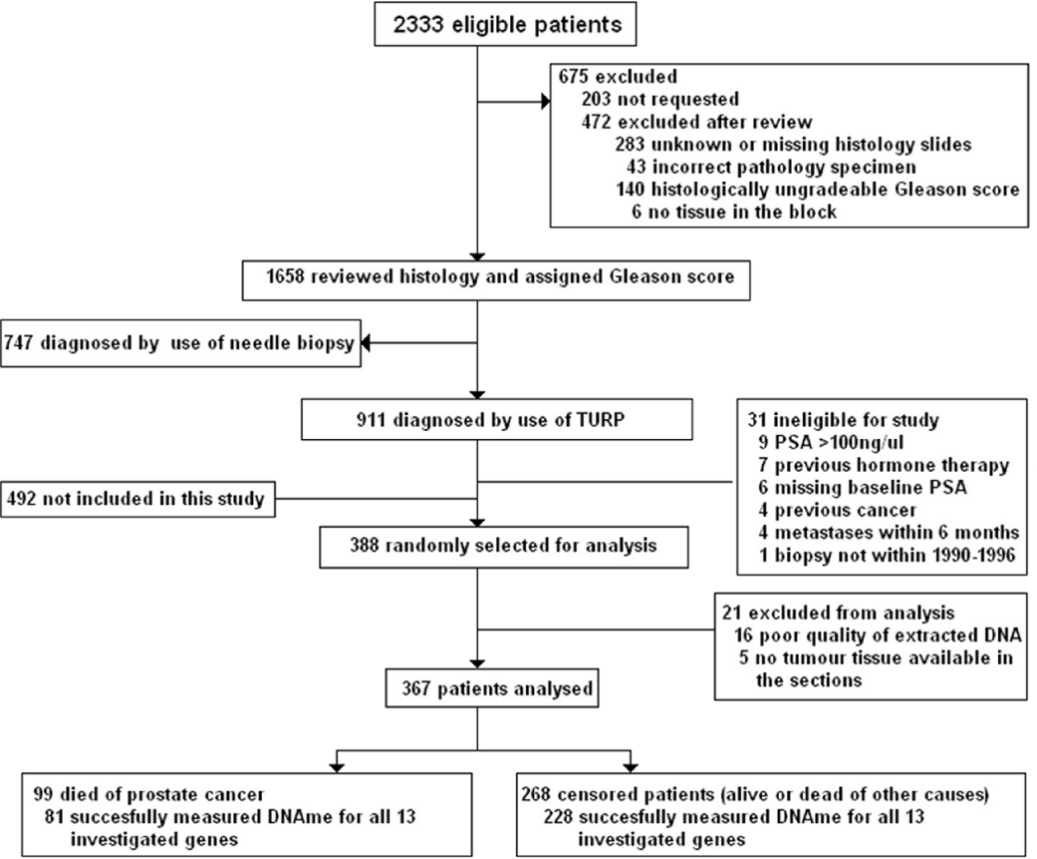
**Consort diagram of TAPG cohort patients enrolled in current study.**

388 formalin-fixed paraffin wax-embedded (FFPE) transurethral resection of prostate (TURP) tissues from the Transatlantic Prostate Group (TAPG) cohort were randomly selected for the current study (Figure  1) [1]. The TAPG cohort comprises well-characterised men residing in the United Kingdom who did not receive any treatment for at least 6 months following diagnosis of prostate cancer. These patients experienced a high rate of prostate cancer-related death and provided sufficient cases to establish our endpoint of interest. Briefly, FFPE prostate cancer tissue blocks were obtained from six cancer registries in Great Britain. Men were included if they had clinically localised prostate cancer diagnosed by TURP between 1990 and 1996 inclusive, and were younger than 76 years at the time of diagnosis. To focus on patients likely to have biologically localised disease at presentation - patients were excluded if 1) treated by radical prostatectomy, hormones, radio- or chemotherapy 2) showed objective evidence of metastatic disease and 3) had a PSA measurement above 100 ng/ml. Patients who died at or within 6 months of diagnosis were automatically excluded. Following triage by a single expert prostate pathologist (DMB) the original histological TURP specimens were reviewed by a panel of expert urological pathologists to confirm the diagnosis and, when necessary, to reassign scores by use of a contemporary interpretation of the Gleason scoring system [24]. The primary endpoint was death from prostate cancer and outcomes were determined through medical records and cancer registry records. Where available, death certificates were reviewed to verify cause of death. Deaths were divided into two categories: death from prostate cancer and death from other causes, according to standardised World Health Organisation criteria [25]. Patients still alive at last follow-up in December 2009 were censored.

National ethics approval was obtained from the Northern Multicentre Research Ethics Committee, followed by local ethics committee approval at each of the collaborating NHS hospital trusts (Ashford & St. Peter’s, Barnet & Chase Farm, Brighton and Sussex, Dartford & Gravesham, East & North Hertfordshire, Eastbourne, Epsom & St. Helier, Essex Rivers Healthcare, Frimley Park, Greenwich Healthcare, Guy’s & St Thomas’s, Hammersmith Hospitals, Havering Hospitals, Hillingdon, King’s Healthcare, Kingston, Lewisham, Mayday Healthcare, The Medway, Mid Essex Hospitals*;* Mid Kent, North West London Hospitals, Royal Free Hampstead, St Bartholomew’s and The Royal London Hospitals, Royal Surrey County, Southend, St George’s London, St Mary’s London, West Hertfordshire, Worthing & Southlands Hospitals, Airedale, Hull & East Yorkshire, The Leeds Teaching Hospitals, Heatherwood & Wexham Park, Milton Keynes, Northampton, Oxford Radcliffe, Royal Berkshire & Battle, Stoke Mandeville, Ceredigion and Mid Wales, Conwy & Denbighshire, NE Wales, Gwent Healthcare, Swansea, Cardiff & Vale, The Lothian University Hospitals, North Glasgow University Hospitals, Royal Liverpool University Hospital.) [1].

### DNA isolation and bisulfite conversion

FFPE sections were deparaffinised in xylene by submersion two times for 5 minutes and absolute ethanol three times for 5 minutes. From each case an H&E stained section that had been previously annotated for cancerous and normal areas by an expert pathologist (DMB) was used as a guide for macrodissection. Depending on estimated tumour tissue size, one to six 5 μm FFPE sections were dissected [26] and DNA was extracted and converted as previously described [19].

### DNA methylation assay

Our study was conducted following REMARK guidelines [27]. The primer design, sequences and PCR conditions were previously optimised and described [19, 20]. PCRs were performed employing the PyroMark PCR kit (Qiagen, 978703) with standard curves and a converted DNA equivalent of 1000 cells per sample. Presence of the correct amplicons was confirmed by the QIAxcel capillary electrophoresis instrument (Qiagen). Pyromark and PyroGold reagents (Qiagen, 979009, 979006, 972804) were used for the pyrosequencing reaction and the raw pyrogram signals were analysed using the PyroMark Q96 ID system (Qiagen, 9001525) [20].

### Statistical methods

The statistical methods were documented in a pre-specified statistical analysis plan and laboratory testing was blinded from the clinical variables to minimise bias in the results. Three to six CpG positions were analysed per gene and mean methylation of the investigated CpG positions within each assay was used for all analyses. As clinical stage could not be obtained for a significant number of patients, it was completely excluded from our analysis. The Spearman’s rho correlation coefficient was estimated for methylation levels of all gene combinations as well as between each gene and age, PSA score, Gleason score and extent of disease. A univariate Cox regression model with the primary endpoint death from prostate cancer was fitted for each of the available clinical variables and each investigated gene. *P*-values were adjusted for multiple comparisons using the Benjamini Hochberg false discovery rate approach [28]. Stepwise Cox regression models were fitted using all available variables or combination of selected variables to investigate different clinical circumstances and then compared by the likelihood ratio (LR) test. Gene methylation values and clinical variables were analysed as continuous data in all fitted Cox models. The extent of disease estimated from the TURP specimens was excluded in multivariate analysis due to the fact that this variable as defined in our study (percentage of TURP chips with cancer) would either not be available or not be comparable for risk assessment in needle biopsies typical of normal clinical settings.

Kaplan Meier survival curves were plotted for the models presented. All applied tests were two-sided and *P*-values of ≤0.05 were regarded as statistically significant. Statistical analyses were done with STATA 11 and R 2.12.2.

## Results

**Figure 2:**
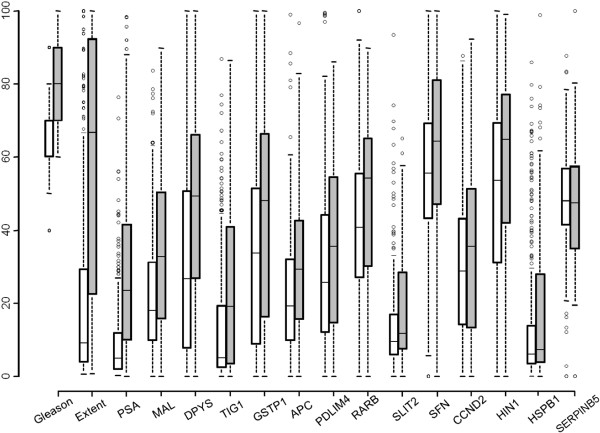
**DNA methylation in two groups of interest.** Comparison and distribution of DNAme percent (y-axis) in each of the investigated genes to the clinical variables in men who died of prostate cancer (grey box) compared to the censored men who were alive at the last visit or died of other causes (white box). Whiskers of the boxplot mark the 5th and 95th percentiles, the box 25th percentile, median and 75 percentile, while extreme values are shown by (•). For graphical presentation, all Gleason score values were scaled by a factor of 10.

DNAme of 13 candidate genes – *GSTP1, APC, RARB, CCND2, SLIT2, SFN, SERPINB5, MAL, DPYS, TIG1, HIN1, PDLIM4* and *HSPB1* was measured in 367 men from the TAPG cohort. 21 patients were excluded after DNA extraction due to no or poor quality tumour DNA obtained (Figure 1). The characteristics of the 367 men are presented in Table 1. Median age was 70.5 years (range 49.9 – 76, IQR = 5.9), median follow-up was 9.5 years (range 0.7-19.6, IQR = 9.2) and there were 99 deaths from prostate cancer. The DNAme measurements for the different genes were of varying success rate (94-99%) (Table 2). The distribution of methylation of each gene was plotted in two groups: men who died of prostate cancer and censored men who were alive at the last visit or had died of other causes (Figure 2).

**Table 1:** **Characteristics of 367 analysed men from TAPG cohort**

Characteristics		No. (%) of patients
All patients		367
DPCa^a^	Yes	99 (27)
	No	268 (73)
Gleason score	Gleason 4	3 (0.8)
	Gleason 5	17 (4.6)
	Gleason 6	171 (46.6)
	Gleason 7	84 (22.9)
	Gleason 8	43 (11.7)
	Gleason 9	43 (11.7)
	Gleason 10	6 (1.6)
PSA score	≤4	138 (37.6)
	4 - ≤10	76 (20.7)
	10 - ≤25	73 (19.9)
	25 - ≤50	54 (14.7)
	50 - 100	26 (7.1)
Extent of Disease	≤0.06	108 (29.4)
	0.06 - ≤0.20	95 (25.9)
	0.20 - ≤0.40	55 (15.0)
	0.40 - ≤0.75	44 (12.0)
	> 0.75	65 (17.7)
Age at diagnosis	≤54	3 (0.8)
	>54 - 64	49 (13.4)
	>64 - 74	253 (68.9)
	>74 - 76	62 (16.9)

^a^DPCa = death from prostate cancer.

**Table 2:** **Univariate Cox regression of 13 genes and available clinical variables**

	HR ^a^ (95% CI)	LR ^b^ χ ^2^	Adjusted ^c^ ***P***-value	Harrell’s c-index	Total No ^d^	Event No ^e^
Gleason score	2.33 (1.99, 2.73)	105.3	2.2*10^-16^	0.79	367	99
Extent of disease	1.27 (1.21, 1.34)	80.1	2.2*10^-16^	0.76	367	99
PSA	1.36 (1.28, 1.45)	68.9	6.3*10^-16^	0.76	367	99
Age	1.04 (1.00, 1.09)	3.2	0.08	0.52	367	99
*MAL*	1.28 (1.17, 1.40)	25.4	2.0*10^-6^	0.64	352	95
*DPYS*	1.20 (1.12, 1.29)	24.2	2.9*10^-6^	0.65	344	95
*TIG1*	1.25 (1.14, 1.36)	20.9	1.4*10^-5^	0.65	350	90
*GSTP1*	1.17 (1.08, 1.26)	16.4	1.2*10^-4^	0.62	357	98
*APC*	1.18 (1.08, 1.29)	10.9	0.002	0.61	365	99
*PDLIM4*	1.16 (1.06, 1.26)	10.9	0.002	0.60	365	98
*RARB*	1.13 (1.04, 1.24)	7.7	0.01	0.60	351	98
*SLIT2*	1.17 (1.05, 1.31)	6.6	0.016	0.58	350	94
*SFN*	1.13 (1.02, 1.25)	5.8	0.023	0.57	363	99
*CCND2*	1.12 (1.02, 1.23)	5.2	0.029	0.56	364	99
*HIN1*	1.09 (1.01, 1.18)	5.1	0.029	0.59	350	97
*HSPB1*	1.12 (1.02, 1.22)	5.0	0.029	0.52	349	91
*SERPINB5*	0.95 (0.83, 1.08)	0.7	0.408	0.53	357	95

^a^The hazard ratios were calculated per 10 units increase in age, PSA, extent of disease and gene methylation while it is per unit increase in Gleason score, i.e. 4 through 10.

^b^LR = likelihood ratio test.

^c^The Benjamin and Hochberg step-up procedure for controlling false discovery rate (FDR) was applied with FDR of 5%.

^d^The total number of patients for which DNAme was successfully measured. The clinical variables were available for all men included in the study.

^e^The number of patients for which a DNAme result was obtained and who died of prostate cancer.

Univariately, methylation of 12 genes was associated to prostate cancer-specific death (Table 2). Gleason score was the strongest predictor with the hazard ratio (HR) 2.33 [95% CI 1.99-2.74] for each unit increment (i.e. Gleason score 4, 5, …10). In comparison, the strongest among genes, *MAL* displayed HR 1.28 [95% CI 1.17-1.40] per 10% increment in methylation (Table 2). To make clinical variables more comparable to DNAme, the HR for the PSA (ng/mL), extent of disease (%) and age (year) were also calculated per 10 unit increments.

Methylation was successfully measured for all 13 genes in 309 patients including 81 prostate cancer-specific deaths and this subset was used for the stepwise multivariate Cox regression models. To assess clinical utility of DNAme, mortality prediction by models investigating four distinct sets of variables were considered: A) Methylation of 13 genes, B) Molecular variables (gene methylation and PSA), C) Current clinical standard (Gleason score and PSA) and D) All variables (including the interaction between the gene methylation and the clinical variables). Model D was the best multivariate model with LR χ^2^_(6df)_ = 125.7, which included Gleason score, PSA, *DPYS, HSPB1*, interaction term [HSPB1xGleason score] and *CCND2* (Table 3). In comparison, model C was the next best model with LR χ^2^_(2df)_ = 111.4. Model B was formed of PSA and methylation of *DPYS*, *HSPB1*, *MAL* and *TIG1* with LR χ^2^_(5df)_ = 76 and the gene-only model comprised: *DPYS*, *GSTP1*, and *MAL* with LR χ^2^_(3df)_ = 49.4 (Table 3). As a higher likelihood ratio χ^2^ indicates a better model. The Δχ^2^_(4df)_ between model D and C was 14.3 (*P* =0.006), which shows that a set of variables corresponding to differential DNA methylation of the identified genes adds a statistically significant amount of information to the risk prediction of current clinical reference standard (Table 3).

**Table 3:** **Multivariate Cox models**

	Model A:Gene-Only			Model B:Genes + PSA			Model C:Gleason + PSA			Model D:Final model		
Variable	HR (95% CI)	χ ^2^	***P***-value	HR (95% CI)	χ ^2^	***P***-value	HR (95% CI)	χ ^2^	***P***-value	HR (95% CI)	χ ^2^	***P***-value
**Gleason**	-^b^	-	-	-	-	-	2.20 (1.82, 2.67)	66.3	3.3*10^-16^	2.72 (2.09, 3.53)	56.3	6.4*10^-14^
**PSA**	-	-	-	1.27 (1.18, 1.38)	36.5	1.5*10^-9^	1.27 (1.17, 1.37)	34.9	3.5*10^-9^	1.23 (1.13, 1.34)	24.7	6.7*10^-7^
***DPYS***	1.12 (1.02, 1.24)	5.8	0.016	1.12 (1.02, 1.24)	5.3	0.021	-	-	-	1.13 (1.03, 1.25)	6.4	0.012
***HSPB1***				0.88 (0.78, 0.99)	4.6	0.032	-	-	-	2.39 (1.15, 4.97)	5.5	0.019
**Gleason x** ***HSPB1*** ^**a**^	-	-	-	-	-	-	-	-	-	0.89 (0.81, 0.97)	6.2	0.012
***CCND2***							-	-	-	0.86 (0.75, 0.98)	5.1	0.024
***MAL***	1.19 (1.05, 1.34)	7.6	0.006	1.17 (1.03, 1.34)	5.7	0.017	-	-	-			
***GSTP1***	1.15 (1.03, 1.27)	6.6	0.010				-	-	-			
***TIG1***				1.15 (1.03, 1.27)	6.5	0.011	-	-	-			
**LR χ** ^**2**^ **(df)**	49.4 (3)			76.6 (5)			111.4 (2)			125.6 (6)		
**Harrell’s c-index (se)**	0.716 (0.034)			0.771 (0.034)			0.831 (0.034)			0.835 (0.034)		
**Gönen & Heller’s c-index** ^**c**^ **(se)**	0.696 (0.022)			0.702 (0.019)			0.738 (0.016)			0.757 (0.017)		

^a)^Cross-product of Gleason score multiplied by *HSPB1* methylation. For construction of a full model, all clinical variables and genes were included as well as interaction terms between each of the genes and the variables. The only significant interaction was found for Gleason score and *HSPB1*.

^b)^Variable not included in model.

^c)^The G**ö**nen & Heller’s c-index is independent of the degree of censoring and is somewhat comparable to an area under the curve corresponding to a plot of the sensitivity versus positive predictive value of the predictor.

(df) = degrees of freedom.

(se) = standard error.

**Figure 3:**
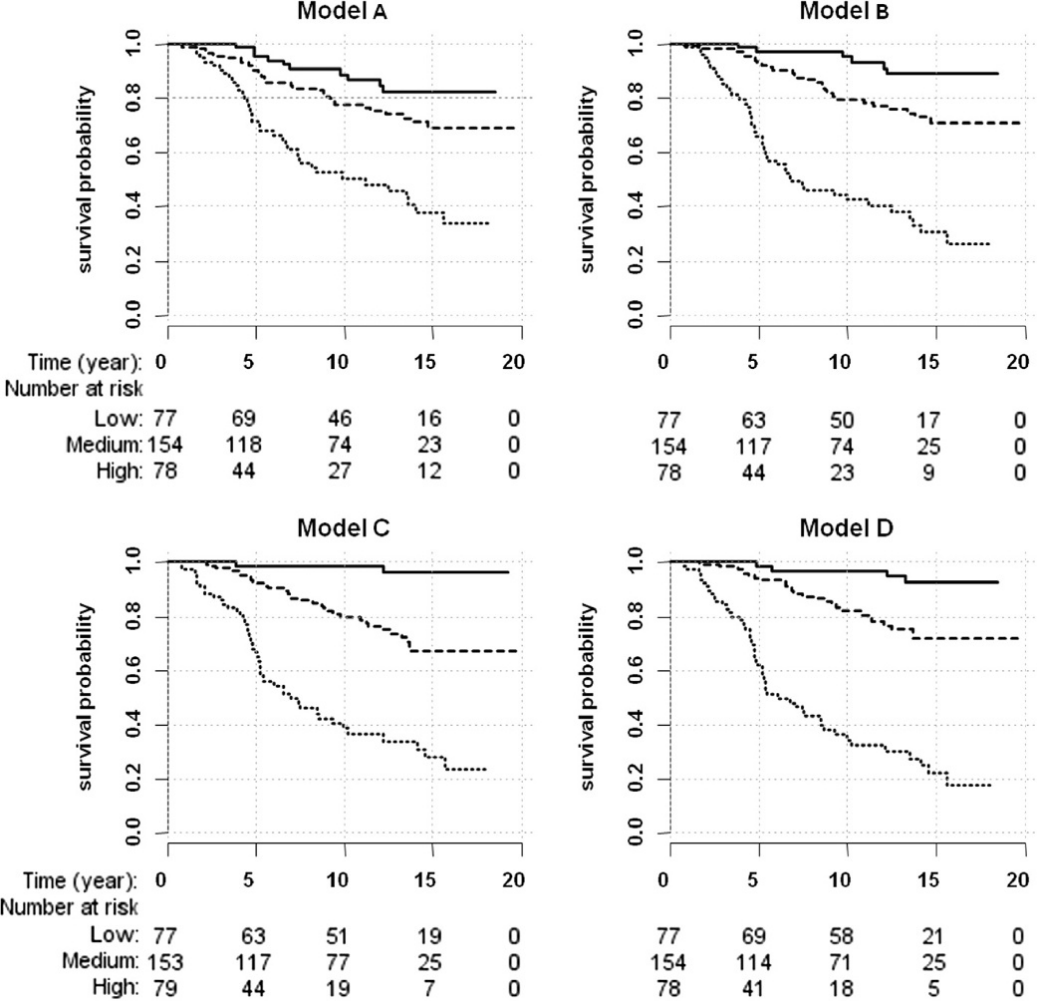
**Kaplan Meier survival analysis curves for the fitted models. A)** *DPYS*, *GSTP1* and MAL, **B)** PSA and DNAme of *DPYS*, *HSPB1*, *MAL* and *TIG1*, **C)** Gleason score and PSA and **D)** the full model with Gleason score, PSA, *DPYS, HSPB1*, [HSPB1xGleason score] and *CCND2*. Low (solid line), medium (dashed line) and high risk groups (dotted line) were separated by the 25% and 75% quantiles.

The risk scores obtained from the linear predictors of the four models were categorised into low, medium and high risk groups using the 25% and 75% quantiles and Kaplan Meier survivor curves were plotted (Figure 3). The proportion of prostate cancer-specific deaths in each of the groups low, median and high were calculated for the different models (Additional file 1: Table S1) expanding the information from the curves. Kaplan Meier survivor curves illustrated that although the models including Gleason score are best, use of PSA in combination with gene methylation provided a similar amount of information, particularly for identifying patients at highest risk (Figure 3B).

To explore the effect of competing risks we fitted a proportional hazards model which assesses the effect of covariates on the sub-distribution of a particular type of failure in a competing risks setting (performed by means of the R-package cmprsk). A stepwise model selection analysis was performed, yielding the same markers that were selected by stepwise model selection using an ordinary Cox model (data not shown).

As an internal validation of the improvement of model D compared to model C, intended to correct for statistical optimism, we used the original data (n = 309, excluding missing values) on survival time, event and predictors. Models were fitted in the bootstrap sample (with replacement) and a backward stepwise method was applied at significance level 0.05 for a predictor to be kept in a model. The final selected Cox model was fitted in the bootstrap sample and applied without change to the original sample. The process was repeated for B = 1000 bootstrap replications to obtain an average optimism, which was subtracted from the fit value of the final models [29]. We were primarily interested in the resulting optimism corrected G**ö**nen & Heller’s c-index because this index is independent of the degree of censoring and more accurately reflects diagnostically important differences; the c-index for Model C was 0.737 and for model D was 0.741, showing an internally validated small improvement for a classifier that includes the DNA methylation biomarkers.

## Discussion

This study has revealed several biomarkers of promising prognostic value in prostate cancer following measurement of the methylation of particular gene promoters/first exons. In the univariate analysis, 12 of the 13 investigated genes with HR ranging between 1.09 and 1.28 per a decile increase in DNAme (Table 2) were significantly associated to prostate cancer-specific death. While Gleason score by specialist prostate pathologists employing strict criteria remained the best available prognostic variable (LR χ^2^ = 105.3), morphological appearance is a vectorial parameter resulting from the interaction of several individual key genes or their products that contribute significantly to clinical outcome. In the comprehensive multivariate analysis, the model with best prognostic ability included Gleason score, PSA, *HSPB1*, [HSPB1xGleason score], *CCND2* and *DPYS* (Table 3) demonstrating that gene methylation added significant information for predicting prostate cancer-related death. In contrast to univariate analysis, where methylation of *MAL* was most prognostic amongst genes (LR χ^2^ = 25.4), *MAL* was not selected in the final multivariate model. Plausibly, this reflects the strong correlation of methylation of *MAL* to both Gleason score and PSA. A variable that appears strong in univariate analysis would be eliminated in a multivariate analysis by a stronger variable if it adds similar information to the model due to strong correlation. Further, this can explain the difference between our results regarding the prognostic biomarker potential of *APC* and *GSTP1* and a previous study where prostate cancer-specific death was also the primary endpoint [21]. Other factors contributing to the discrepancy may be utilisation of different methods for assessment of methylation as well as a different repertoire of clinical variables.

Enhanced expression of protein HSP27 encoded by the gene *HSPB1* was earlier shown to be a reliable biomarker of poor-outcome cancers [30, 31]. Recently, we reported that *HSPB1* methylation and its interaction with Gleason score has prognostic value and may be of clinical importance for risk stratification of men in the low risk (<7) Gleason score group [19]. Here, in a multivariate comparison with 12 other genes, *HSPB1* methylation and its interaction term with Gleason score remained important for risk stratification (Table 3).

Similarly to *HSPB1, CCND2* methylation displayed an HR of 0.86 [95% CI 0.75-0.98] (Table 3) indicating that higher levels of methylation were associated to lower risk of prostate cancer death, consistent with the role of activated *CCND2* as an oncogene. Previously, the prognostic value of *CCND2* had been evaluated only with respect to biochemical reoccurrence and with discordant findings [22, 32].

*DPYS* appeared useful for predicting prostate cancer-specific mortality in all models where gene methylation was included (Table 3). Furthermore, the distribution of methylation showed the largest difference in median methylation between the two groups of patients (Figure 2). Although aberrant methylation of *DPYS* has been reported by us and others [20, 33] this is the first report demonstrating its prognostic value in prostate cancer.

Extensive research efforts have suggested a number of candidate biomarkers and biomarker panels, including PCA3 [34], TMPRSS-ERG [35], Ki-67 [36], and CCP score [37] to improve the clinical management of prostate cancer. Ideally, a biomarker detected by molecular testing of bodily fluids is necessary to avoid intrusive examinations and potentially harmful biopsies. Therefore, we compared differences in survival prediction capabilities between a model based on the current clinical reference standard and models that excluded Gleason score but were based on PSA and molecular epigenetic variables that may be obtained from a serum or urine test. A model including PSA, and methylation of *DPYS*, *HSPB1*, *MAL* and *TIG1* was better at predicting prostate cancer-related mortality than a model based only on gene methylation (Table 3). Significance of *TIG1* methylation for mortality prediction was identified only in the absence of Gleason score, probably because of the strong correlation between these variables. A recent report supports the prognostic value of *TIG1* methylation [23]. Comparing the PSA-Gleason score with PSA-gene methylation model, a similar proportion of men were classed in the low, medium and high risk groups (Figure 3). Furthermore, the proportion of men who died in each of the groups (Additional file 1: Table S1) showed a modest decrease in sensitivity of the PSA-gene model compared to the PSA-Gleason model; however, specificity was similar, thus prompting future efforts to assessment of DNA methylation in body fluids. Although TURP is not the standard modality for the diagnosis of prostate cancer, the use of TAPG TURP specimens allowed us to assemble a unique cohort of untreated men with prostate cancer with up to 20 years of follow-up and thereby study the association of DNA methylation to death from prostate cancer. To eliminate any potential bias introduced by use of TURP tissues, validation of the current PSA and gene methylation model is needed in a cohort comprising of needle biopsies.

## Conclusions

Multivariate analysis indicated that methylation of genes *DPYS*, *CCND2* and *HSPB1* added significant prognostic information and may allow more accurate prediction of men who can be safely managed by active surveillance. Also, development of a test based upon methylation of *DPYS*, *HSPB1*, *MAL* and *TIG1* complementing use of PSA may improve identification of men who require a biopsy. Assays measuring methylation of *MAL, TIG1, HSPB1, CCND2,* and *DPYS* have potential to accurately stratify early prostate cancers and thereafter to manage affected patients in a biologically appropriate manner.

## Electronic supplementary material

Additional file 1: Table S1: Proportion of death in the groups low, medium and high as shown in Figure 3 and prediction value of different models. (DOC 30 KB)

## Abbreviations

PSA: Prostate specific antigen
DNAme: DNA methylation
FFPE: Formalin-fixed paraffin-embedded
TAPG: Transatlantic Prostate Group
TURP: Transurethral resection of prostate
HR: Hazard ratio
LR: Likelihood ratio
DPCa: Death from prostate cancer
PCR: Polymerase chain reaction.
