# Supplementary material for: DNA methylation gene-based models indicating independent poor outcome in prostate cancer
Source: BMC Cancer. 2014 Sep 6;14:655. doi: 10.1186/1471-2407-14-655 (PMC4162944; doi:10.1186/1471-2407-14-655)
Supplement: Supplementary file 1 — Additional file 1: Table S1: Proportion of death in the groups low, medium and high as shown in Figure 3 and prediction value of different models. (DOC 30 KB) [file 12885_2014_4830_MOESM1_ESM.doc]

Supplementary Material

Additional file 1: Table S1. Proportion of death in the groups low, medium and high as shown in Figure 3 and prediction value of different models.

| **Model^a^** | **χ²** | **Low** | **Medium** | **High** |
| --- | --- | --- | --- | --- |
| A: genes only | 49.354 | 13% | 22% | 47% |
| B: PSA + genes | 76.598 | 8% | 20% | 58% |
| C: Gleason + PSA | 111.441 | 3% | 22% | 58% |
| D: Final model ^b)^ | 125.646 | 5% | 18% | 64% |

^a)^ All models have *P*-value < 0.0001

^b)^ Model including Gleason score, PSA, HSPB1xGleason score interaction term and methylation of *DPYS, HSPB1*, and *CCND2*.
